# Choosing Important Health Outcomes for Comparative Effectiveness Research: An Updated Review and Identification of Gaps

**DOI:** 10.1371/journal.pone.0168403

**Published:** 2016-12-14

**Authors:** Sarah L. Gorst, Elizabeth Gargon, Mike Clarke, Valerie Smith, Paula R. Williamson

**Affiliations:** 1 MRC North West Hub for Trials Methodology Research (NWHTMR), Department of Biostatistics, University of Liverpool, Liverpool, United Kingdom; 2 Northern Ireland Network for Trials Methodology Research, Queen's University Belfast, Belfast, United Kingdom; 3 School of Nursing & Midwifery, Trinity College Dublin, Dublin, Ireland; Johns Hopkins University Bloomberg School of Public Health, UNITED STATES

## Abstract

**Background:**

The COMET (Core Outcome Measures in Effectiveness Trials) Initiative promotes the development and application of core outcome sets (COS), including relevant studies in an online database. In order to keep the database current, an annual search of the literature is undertaken. This study aimed to update a previous systematic review, in order to identify any further studies where a COS has been developed. Furthermore, no prioritization for COS development has previously been undertaken, therefore this study also aimed to identify COS relevant to the world’s most prevalent health conditions.

**Methods:**

The methods used in this updated review followed the same approach used in the original review and the previous update. A survey was also sent to the corresponding authors of COS identified for inclusion in this review, to ascertain what lessons they had learnt from developing their COS. Additionally, the COMET database was searched to identify COS that might be relevant to the conditions with the highest global prevalence.

**Results:**

Twenty-five reports relating to 22 new studies were eligible for inclusion in the review. Further improvements were identified in relation to the description of the scope of the COS, use of the Delphi technique, and the inclusion of patient participants within the development process. Additionally, 33 published and ongoing COS were identified for 13 of the world’s most prevalent conditions.

**Conclusion:**

The development of a reporting guideline and minimum standards should contribute towards future improvements in development and reporting of COS. This study has also described a first approach to identifying gaps in existing COS, and to priority setting in this area. Important gaps have been identified, on the basis of global burden of disease, and the development and application of COS in these areas should be considered a priority.

## Introduction

Comparative effectiveness research (CER) involves comparing the benefits and harms of interventions for clinical conditions, which will assist patients, providers, and policy makers in formulating informed decisions that will improve health care [1]. One important element of CER is to ensure that appropriate outcomes are measured in research, so that findings can be compared and contrasted across different studies, and useful evidence can be provided to decision makers. Core outcome sets (COS) will help to achieve this. COS represent an agreed set of outcomes that should be measured and reported, as a minimum, in all clinical trials for a specific health condition [2]. The application of COS allows the results of clinical trials to be appropriately combined, minimizing waste [3] and ensuring that usable evidence is made available.

The COMET (Core Outcome Measures in Effectiveness Trials) Initiative (www.comet-initiative.org) promotes the development and application of COS, by including pertinent individual studies in a publically available online database. In 2013, the first comprehensive search for COS in health research was conducted to ensure that the database content was comprehensive and up-to-date [4]. This review was updated in 2015 [5] and demonstrated that studies appeared to have adopted a more structured approach towards COS development. Additionally, the review highlighted an increase in patient participation in the development process. When using the term ‘patient participation’ throughout this report we are referring to the patients who participated in the development of the COS by contributing to the results of the study, i.e. completing a survey, taking part in interviews, attending consensus meetings, etc.

A previous survey [5] shows that people are checking the COMET database to see whether a COS exists in their area of interest to avoid duplication and to inform the outcomes collected in trials. This emphasises the importance of keeping the database current. There is also a need to identify health areas that are in particular need of a COS [6]. If COS were available and utilized for the most prevalent health conditions, this should accelerate the impact of research and result in improvements in global health.

### Aims

The aims of the current study were to (i) update the systematic review [4,5], in order to identify any further studies where a COS has been developed; (ii) ascertain what lessons have been learnt from developing COS; and (iii) identify published and ongoing COS that are relevant to the most prevalent health conditions throughout the world, to identify important areas where there are a lack of COS, and to highlight areas for future COS development or improvement.

## Methods

### Systematic review update

The methods used in this updated review followed the same approach used in the original review and the previous update [4,5].

### Study selection

#### Inclusion and exclusion criteria

As described in detail previously [4], studies were eligible for inclusion if they had applied methodology for determining which outcome domains or outcomes should be measured, or are important to measure, in clinical trials or other forms of health research. By using the term ‘outcome’ we are referring to something that occurs as a result of the specific health condition (e.g. diarrhoea) and by ‘outcome domain’ we are referring to the grouping of individual outcomes (e.g. bowel function, which would include diarrhoea).

#### Types of participants and interventions

As previously [4], studies were categorised as eligible if they reported the development of a COS, regardless of any restrictions by age, health condition or setting, which could be used to assess the effect of interventions for that condition.

### Identification of relevant studies

In January 2016, we searched MEDLINE via Ovid and SCOPUS (including EMBASE) without language restrictions. The search identified studies that had been published or indexed between the previous systematic review update [5] in January 2015 and the end of December 2015. The multifaceted search strategy developed in the original review using a combination of text words and index terms [7] was used in the current review, with adaptations appropriate for each database (S1 Table). In addition to this database searching, we also completed hand searching activities. We identified any studies that had been directly submitted to the COMET database. We also examined references cited in eligible studies and in ineligible studies that referred to or used a COS.

### Selecting studies for inclusion in the review

Records from each database were combined and duplicates were removed. Titles and abstracts were read to assess eligibility of studies for inclusion in the review (stage 1). Full texts of potentially relevant articles were obtained to assess for inclusion (stage 2). Two of three reviewers (SG, VS and EG) independently checked the title and abstract of each citation. Citations were retained for further checking if agreement could not be reached. One reviewer (SG) assessed each full paper for inclusion in the review and two reviewers (VS and EG) each assessed half of the full papers. Reasons for exclusion at this stage were documented for articles judged to be ineligible.

### Checking for agreement between reviewers

During each stage of the review process, agreement between reviewers was assessed. Prior to independently assessing records, the three reviewers (SG, VS and EG) independently checked batches of abstracts and full papers for agreement.

### Checking for correct exclusion

Of the records that had been excluded on the basis of the title and abstract, full text papers were obtained for a 1% sample and a fourth reviewer (Jennifer Weston) assessed correct exclusion. If any studies were identified as being incorrectly excluded, further checking was performed within the other excluded records. Of the records that had been excluded after reading their full text papers, 5% were assessed for correct exclusion at that stage.

### Data extraction

As described in detail previously [4], data were extracted in relation to the study aims, health area, target population, methods of COS development and stakeholder groups involved.

### Data analysis and presentation of results

The results are presented descriptively.

### COS developer survey

In May 2016, an email was sent to the corresponding authors of new COS identified for inclusion in this systematic review. The email informed developers that their study had been identified for inclusion in the review and asked them to respond to one question: ‘If I had known then what I do now, what would I have done differently?’ The email explained that we would like to find out what lessons they had learnt from developing their COS and whether they had any advice for future developers. The results of the survey were analyzed thematically to identify themes within the developers’ responses [8]. Survey responses were coded and collated to identify significant broader patterns of meaning, which transpired into potential themes. The viability of each of these potential themes was then reviewed and refined alongside the original survey responses to ensure that they answered the research question, ‘If I had known then what I do now, what would I have done differently?’

### Priority setting

The COMET database was searched (June 2016) to identify published and ongoing COS relevant to the 25 conditions with the highest global prevalence identified in the Global Burden of Disease Study [9]. Details of any relevant COS were extracted in relation to health condition, year of publication, target population, intervention, setting, and participating stakeholder groups and countries.

## Results

### Description of studies

**Fig 1:**
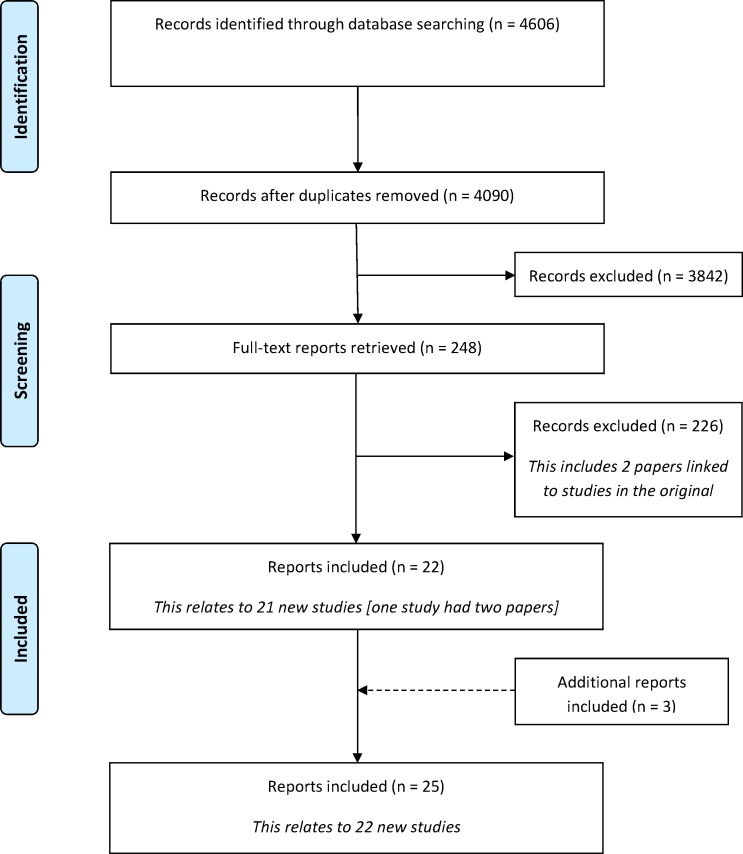
Identification of studies.

Following the removal of duplicates, 4090 citations were identified in the database search. A total of 3842 records were excluded during the title and abstract stage, and a further 229 were excluded following the assessment of full text papers (Fig 1). S2 Table provides a summary of the reasons for exclusion of the full text papers. Twenty-two citations related to 21 new studies met the inclusion criteria. In addition to the database search, three additional citations were identified as being eligible for inclusion in the review. Two of these were identified as being linked papers to one of the included studies and a further study was identified following a submission to the COMET database by the study authors. In total, 25 reports relating to 22 new studies were included for the first time in this update (S3 Table).

### Included studies

#### Year of publication

**Fig 2:**
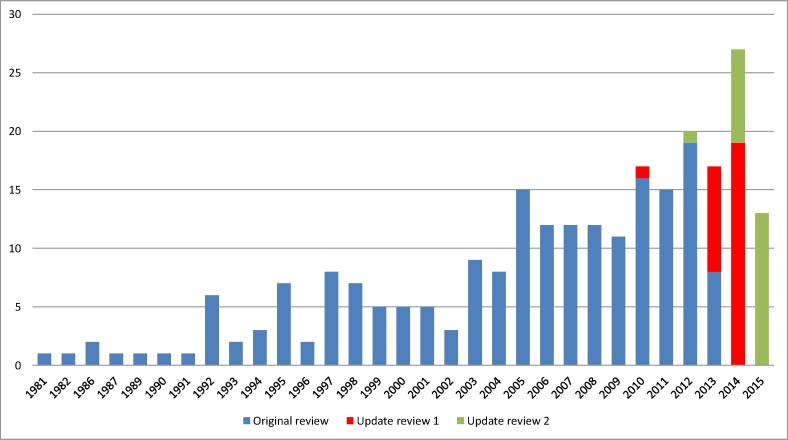
Year of first publication of each COS study (n = 249).

Our analysis of the year of first publication of each COS included in the previous reviews has been updated to include the 22 new studies identified in this updated review (Fig 2). Of the 22 studies identified in this update, 21 studies were published between 2014 and 2015, and one study was published in 2012. This study was identified through the database submission.

#### Scope of core outcome sets

The scope of published COS studies is summarised in Table 1 and includes the 227 COS that were included in the two previous systematic reviews and the 22 new COS that have been added by this updated review. This includes study aims, setting for intended use, population characteristics and intervention characteristics.

**Table 1:** The scope of included studies (n = 249).

	Original review n (%)	Update review 1 n (%)	Update review 2 n (%)	Combined n (%)
**Number of included COS studies**	198	29	22	249
**Study aims**				
Specifically considered outcome selection and measurement	97 (49)	22 (76)	15 (68)	134 (54)
Considered outcomes while addressing wider clinical trial design issues	101 (51)	7 (24)	7 (32)	115 (46)
**Intended use of recommendations**				
Clinical trials	141 (71)	19 (66)	16 (73)	176 (71)
Clinical research	27 (14)	4 (14)	4 (18)	34 (14)
Clinical research and practice	11 (6)	4 (14)	1 (5)	16 (6)
Clinical trials and clinical practice	10 (5)	0 (0)	0 (0)	10 (4)
Clinical trials and regulatory purposes	3 (2)	0 (0)	0 (0)	4 (2)
Clinical trials and observational studies	3 (2)	0 (0)	1 (5)	4 (2)
Clinical trial extension studies		1 (4)	0 (0)	1 (<1)
Clinical trials, research and clinical record keeping		1 (4)	0 (0)	1 (<1)
Observational studies	1 (<1)	0 (0)	0 (0)	1 (<1)
Clinical trials and case series	1 (<1)	0 (0)	0 (0)	1 (<1)
Clinical research, clinical practice and regulatory purpose	1 (<1)	0 (0)	0 (0)	1 (<1)
**Population characteristics**				
Adults	10 (5)	11 (38)	6 (27)	27 (11)
Children	23 (12)	2 (7)	6 (27)	31 (12)
Adults and children	13 (7)	1 (4)	0 (0)	14 (6)
Older adults	3 (2)	1 (4)	0 (0)	4 (2)
Adults and neonates	0 (0)	1 (4)	0 (0)	1 (<1)
Not specified	149 (75)	13 (45)	10 (46)	172 (69)
**Intervention characteristics**				
All intervention types	7 (4)	9 (31)	12 (55)	28 (11)
Drug treatments	40 (20)	4 (14)	0 (0)	44 (18)
Surgery	13 (7)	4 (14)	6 (27)	23 (9)
*Surgery only*	*13*	*2*	*4*	
*Surgery and compression therapy*	*0*	*1*	*0*	
*Surgery and injection*	*0*	*1*	*0*	
*Surgery and device*	*0*	*0*	*1*	
Surgery and conservative management	*0*	*0*	*1*	
Vaccine	2 (1)	0 (0)	0	2 (1)
Rehabilitation	1 (1)	1 (4)	0	2 (1)
Exercise	1 (1)	1 (4)	1 (5)	3 (1)
*Exercise (physical activity)*	*1*	*0*	*1*	
*Exercise (yoga)*	*0*	*1*	*0*	
Procedure	5 (3)	0 (0)	2 (9)	7 (3)
Device	3 (2)	0 (0)	0 (0)	3 (1)
Other	11 (6)	5 (17)	0 (0)	16 (6)
Not specified	115 (58)	5 (17)	1 (5)	121 (49)

#### Methods used to select outcomes

The methods used to develop the 22 new COS identified in the current review are presented in Table 2 alongside the methods used in the two previous systematic reviews [4,5]. Table 2 highlights that there has been an increase in the use of mixed methods to develop a COS, rising from 37% of studies included in the original systematic review to 64% of studies included in this updated review. There has been an increase in the use of the Delphi technique in particular, with 55% of studies included in this updated review using the Delphi technique either alone or alongside other methods.

**Table 2:** The methods used to develop core outcome sets (n = 249).

Main methods	Original review n (%)	Update review 1 n (%)	Update review 2 n (%)	Combined n (%)
Semi-structured group discussion only	57 (29)	2 (7)	2 (9)	61 (25)
Unstructured group discussion only	18 (9)			18 (7)
Consensus development conference only	12 (6)		1 (5)	13 (5)
Literature/systematic review only	11 (6)	5 (17)	2 (9)	18 (7)
Delphi only	6 (3)	2 (7)	2 (9)	10 (4)
Survey only	3 (2)			3 (1)
NGT only	1 (1)			1 (<1)
Mixed methods *(see descriptions below*)	74 (37)	18 (62)	14 (64)	106 (43)
*Delphi + another method(s)*	*23 (12)*	*7 (24)*	*10 (46)*	*40 (16)*
*Semi-structured group discussion + another method(s)*	*29 (15)*	*7 (24)*	*4 (18)*	*40 (16)*
*Consensus development conference + another method(s)*	*7 (4)*			*7 (3)*
*Literature/systematic review + another method(s)*	*10 (5)*	*4 (14)*		*14 (6)*
*NGT + another method(s)*	*4 (2)*			*4 (2)*
*Focus group + another method(s)*	*1 (1)*			*1 (<1)*
No methods described	16 (8)	2 (7)	1 (5)	19 (8)

#### People involved in selecting outcomes

S5 Table lists the stakeholders that were involved in selecting outcomes for inclusion in the COS identified in the previous reviews and this update. In regards to the 249 published COS studies, 214 have provided details about the stakeholders who participated in the development process. Of these 214 studies, clinical experts have been involved in selecting outcomes for inclusion in 212 studies; this is in contrast to patients who have been included in only 55 studies. The rate of inclusion of patient representatives has, however, increased from 18% of studies included in the original systematic review (n = 31/174) to 61% of studies in this updated review (n = 11/18). The degree of patient participation within the development of the COS studies included in this updated review is described in Table 3. Other than stating that patients did participate in the development of the COS, three studies did not provide any further details relating to the degree of participation. The geographical locations of participants involved in developing the COS included in this update and the two previous reviews are shown in S6 Table.

**Table 3:** Patient participation detail where reported (n = 8).

	Methods used	Total number of participants	Number of patient/public participants	% Patient/public participants
1	Delphi (mixed)	Round 1: 143	Round 1: 10	7%
		Round 2: 130	Round 2: 8	6%
		Round 3: 117	Round 3: 9	8%
2	Delphi (mixed)	Round 1: 43	Round 1: 4	9%
		Round 2: 37	Round 2: 3	8%
		Round 3: 39	Round 3: 3	8%
		Round 4: 36	Round 4: 3	8%
3	Interviews (patient only)	65	65	
	Survey (patient only)	51	51	
	Delphi (clinician only)	Round 1: 104	Round 1: 0	
		Round 2: 85	Round 2: 0	
		Round 3: 73	Round 3: 0	
	Meeting (mixed)	14	3	21%
4	Delphi (clinician only)	Round 1: 70	Round 1: 0	
		Round 2: 56	Round 2: 0	
	Delphi (patient only)	Round 1: 31	Round 1: 31	
		Round 2: 32	Round 2: 32	
5	Focus groups (patient only)	97	97	
	Interviews (patient only)	10	10	
	Delphi (clinician only)	Round 1: 233	Round 1: 0	
		Round 2: 232	Round 2: 0	
		Round 3: 227	Round 3: 0	
		Round 4: 191	Round 4: 0	
	Meeting (mixed)	15	8	53%
6	Meeting (mixed	12	3	25%
7	Delphi (mixed)	Round 1: 303	Round 1: 215	71%
		Round 2: 259	Round 2: 190	73%
	Meeting (patient only)	15	15	
	Meeting (clinician only)	23	0	
8	Delphi (mixed)	Round 1: 195	Round 1: 32	16%
		Round 2: 174	Round 2: 25	14%
	Meeting (mixed)	29	2	7%

Table 3 highlights that the methods used, for including patient/public participants within the COS development process, are varied, however the Delphi technique was identified as being the most frequently used method to include patient/public participants. Furthermore, the percentage of patient/public participants included in the COS development process are generally relatively low compared to other stakeholder groups. In contrast, one COS, developed for reconstructive breast surgery, had a 73% patient/public participant inclusion rate. This is much higher than the rates of patient/public participation in all of the other studies. The developers of this particular COS elected to recruit patients and clinicians in a 2:1 ratio to ensure that patients’ views were represented preferentially when the groups were combined, owing to reconstructive breast surgery being a patient-selected optional intervention.

### COS developer survey

Of the 22 COS developers who received the email, eight (36%) responded. Two developers stated that they would not have done anything differently, and provided no further details or advice. The remaining six developers stated what they would have done differently; based on the lessons they had learnt from developing their COS. The responses from the developers have been condensed and summarized (see Table 4).

**Table 4:** COS developer responses to ‘If I had known then what I do now, what would I have done differently?’.

COS developer responses
*Responses from single developer*
Differentiate the 'what' and 'how' at the beginning of the development process
Have a board of people advising you during the process–preferably including all stakeholders
Contact others who have previously developed COS to learn from their experiences
Involve patients from the beginning of the development process
Align development of COS alongside relevant meetings/ conferences/ congresses to improve response rate and speed
Send COS out for wider consultation and review prior to publication
Create an effective way for dissemination, implementation and assessment of the aforementioned tasks
Apply for funding instead of doing it all in own time
*Responses from two developers*
Work closely with COMET and use their experience and ideas to improve and streamline the process
Incorporate more stakeholders of various backgrounds

Stakeholder participation was a dominant theme amongst COS developer responses. Many developers spoke about the importance of including a diverse range and unrestricted number of stakeholders. One developer mentioned that in addition to including disease experts *“it would also be beneficial to take into account the opinions of doctors who wouldn't be deemed to be leaders in the field or key opinion leaders i*.*e*. *GPs… as they often come cross patients… and must manage these cases”* therefore the developer believed it would be interesting *“to see how their view might differ from that of physicians in large referral hospitals”*. Relatedly, one developer stressed the importance of not applying restrictions to the number of stakeholders, stating the need to *“think big and international”*. They suggested inviting every person that is involved in the topic relevant to a COS and then to *“keep emailing people and ask them to appoint 3–5 people that they know to be key leaders in that topic”*. The importance of involving patient stakeholders from the beginning of the COS development process was also mentioned, specifically in the design of patient information material. One developer also had an idea about enhancing the participation of stakeholders in the COS development process, *“by aligning it with [relevant] meetings*, *conferences*, *and congresses”*, as this may help *“to improve overall response rates”* and also the *“speed [of development]”*. The developer claimed that enabling *“COS group members to physically meet and discuss the project [would allow them to] come to a faster and more debate-based decision”*. Relatedly, one developer stated that they *“might have applied for funding instead of doing it all in [their] own time”* as funding may have provided *“more time to involve more stakeholders”*, which would have made their COS *“more representative”* and thus *“better implemented in the future”*.

### Priority setting

A search of the COMET database identified at least one COS for 13 of the 25 conditions with the highest global prevalence (see S7 Table). There are 33 published and ongoing COS that are relevant to 13 of the world’s most prevalent conditions: asymptomatic permanent caries, recurrent tension-type headaches, recurrent migraine, acne vulgaris, low back pain, periodontal diseases, other skin and subcutaneous diseases, asymptomatic deciduous caries, diabetes mellitus, genital prolapse, dermatitis, chronic obstructive pulmonary disease, and chronic hepatitis B infection. Of the 33 COS, 19 are published and 14 are currently ongoing.

More than one COS exists for some conditions as they relate to: (i) a specific type of the condition (e.g. diabetes mellitus: type I diabetes, type II diabetes); (ii) different intervention types (e.g. drugs, behavioural therapies, surgery); (iii) involve different stakeholder groups (e.g. clinical experts, patients, pharmaceutical industry representatives); or (iv) participants from different countries. The majority were, or will be developed with the involvement of people from North America (n = 24; 73%) and Europe (n = 21; 64%). Other geographical locations involved include Australia (n = 4; 12%), Asia (n = 3; 9%), South America (n = 3; 9%), and Africa (n = 1; 3%). Clinical experts have participated in the development of almost all of the 33 COS (n = 31; 94%), with patients participating in 18 (55%).

## Discussion

This study has identified a further 22 studies that determined which outcomes or domains should be measured in all clinical trials for a specific health condition. In regards to the methods used to select outcomes for inclusion in the COS, the use of the Delphi technique for assessing and developing consensus has risen from 15% in the original review to 31% in the previous update and now up to 55% in this current update. This suggests that developers are increasingly adopting a more structured approach to COS development. The Delphi technique allows COS developers to integrate perspectives from multiple stakeholders both anonymously and remotely. Developers may choose to use this method as they see it as means to achieve a higher number of participants, as opposed to some other methods.

Following the previous update, there have been some further improvements in the description of the scope of a COS. In regards to population characteristics, the degree of reporting remained similar to the previous update, with just under half of the studies failing to report population characteristics. However, the reporting of intervention characteristics has increased from 42% of studies included in the original review, to 83% in the previous update, to 95% in the current update. Thus, almost all studies are now clearly defining the intervention(s) that the COS is applicable to. It is clear that developers are reporting more detail about the scope of their COS and it is hoped that this trend will continue, however there remains a need for developers to describe details about the population to which the COS is applicable. A reporting guideline including a checklist of 18 items has recently been developed to facilitate improved reporting of COS [10]. One item requires COS studies to adequately describe both the population and the intervention for which the COS is applicable to.

This article also describes the first approach to identifying gaps in existing COS, and to priority setting in this area. Thirty-three COS (19 published and 14 in development) have been identified for 13 of the world’s most prevalent conditions. No published or ongoing COS have been identified for the remaining 12 most prevalent conditions: iron-deficiency anaemia, glucose-6-phosphate dehydrogenase deficiency trait, age-related and other hearing loss, asymptomatic genital herpes, ascariasis, fungal skin diseases, uncorrected refractive error, trichuriasis, hookworm disease, neck pain, malaria parasitaemia, anaemia, or chronic sequelae, and glucose-6-phosphate dehydrogenase deficiency.

The inclusion of patient participants in the development of COS is particularly relevant for comparative effectiveness research where long term patient centred outcomes are often the important endpoints. The trend towards greater inclusion of patient participants identified in the previous review has further increased in this update, however still over one third of the studies included in this update did not involve patients within the COS development process. Overall, the inclusion of patient participants within the development of COS has now increased to 26%. In regards to the COS that are relevant to the world’s most prevalent conditions, approximately half have included or plan to include patient participants. The lack of patient involvement may trigger additional research in these conditions. The PoPPIE (People and Patient Participation, Involvement and Engagement) Working Group is currently developing resources to assist in the involvement of patient and public representatives within COS development, http://www.comet-initiative.org/resources/publicinvolvement.

COS that are developed globally, i.e. involving stakeholders from multiple locations around the world, could also be deemed to be of greater applicability than those that are developed nationally, if relevant to the specific health condition. The current review identified that Europe and North America continue to be the most predominant locations of participants. Similarly, 82% of COS relevant to the world’s most prevalent conditions, involved participants from Europe and North America, only. Thus, it is evident that COS are failing to include a range of international stakeholders within the development process. However, the inclusion of participants from other continents has increased from 33% in the original systematic review to 55% in this updated review. This is due to the increased participation of stakeholders from Australasia and Asia. The inclusion of stakeholders from South America and Africa is an additional gap that future research should aim to address. However the initial position, for a COS developed using rigorous methodology, should be to ask why the COS would not apply to the particular country of interest, rather than requiring a COS to have involved participants from that country, before it could be adopted.

### Implications

The 22 COS studies that have been identified in this updated review have been added to the COMET database, thus ensuring the ready availability of the most up-to-date COS. This will continue to assist researchers, clinicians and other relevant stakeholders when designing clinical trials, clinical guidelines, and systematic reviews. Additionally, it will inform people who are thinking about developing a COS, whether one already exists within the area they are interested in, and so it will also help to avoid unnecessary duplication of effort. The COMET database also provides a useful facility for developers to inform the COMET Initiative about the existence of their COS. For example, a study that should have been included as a COS in the original review was excluded at the title and abstract stage then due to an absence of COS related information, and instead has been included in this review, as a result of the study authors submitting it directly to the COMET database. This demonstrates that awareness of the COMET Initiative and the ability for people to register their work within the COMET database is helping to overcome difficulty searching, by increasing the likelihood of the identification of COS.

The results of the COS developer survey highlight the need for COMET to continue working with ongoing developers to provide advice and guidance about how to develop COS. It also supports the attempts of the COMET Initiative to initiate contact between ongoing developers and people who have previously developed COS, to enable ongoing developers to learn about their experiences during the development process. COS developers also emphasised the need to include as many participants as possible and particularly on an international scale. This reflects the findings from the review which highlight the lack of involvement of patients and of people from countries situated outside of Europe and North America.

By identifying the 33 published and ongoing COS that are relevant to 13 of the 25 most prevalent health conditions throughout the world, we have also been able to highlight the fact that there is no existing COS applicable to the remaining 12 conditions. The development and application of COS in these areas would provide the foundation for ensuring that appropriate outcomes are measured and reported in clinical trials for these most prevalent health conditions worldwide. Without such international consensus on the key outcomes for research in these conditions, new studies might not make a full contribution to improving global health and opportunities to reduce waste in research will be lost [3]. Furthermore, a wider range of perspectives, including those of patients, on existing COS are also needed when not otherwise included.

### Limitations

One limitation of the systematic review is that it is designed to provide an update of COS that have been published within the previous year. However, from the studies that have been identified for inclusion in this review, there appears to have been a decrease in the number of studies published in 2015, as compared to the previous five years. This may be because the systematic search was only able to identify studies that had been indexed in the databases between January 2015 and December 2015, therefore studies that are published at the end of 2015 will not become indexed in the database until the following year and so will instead be identified and included in the subsequent update. Thus, it is likely that the number of COS studies published within 2015 will be higher than reported in this paper.

Additionally, there is currently no existing means to establish the quality of the COS reported in this study, however those that involve a wide range of stakeholders, particularly patients, within the development process, would likely be considered to be of higher quality, in comparison to those that include health professionals only. The task of defining quality in a COS is extremely difficult, as each COS can be very different to the rest, due to the range of health conditions, interventions, methods used in development, etc. At present, there is no agreed gold standard approach in which to make a judgement about the quality of the COS, as many COS developers have their own opinions about how a COS should be developed. Therefore, COMET aims to develop minimum standards for COS instead. This work will consider only the ‘minimum standards’ that are considered important when developing a COS, but the intention is that no judgement will be made on the methods/ approaches used to address these standards. The development of these minimum standards will include input from COS developers, patient representatives, and the users of COS e.g. trialists, systematic reviewers, clinical guideline developers. The development is currently in the early stages where an initial list of essential criteria for developing COS is being populated. A data driven multi-stakeholder consensus process will be used to develop the minimum standards for COS.

## Conclusion

In conclusion, we have completed an update of a systematic review of studies which have developed a set of outcomes that should be measured and reported in all clinical trials for a specific health condition. Following the previous review, this update has demonstrated an increase in the number of studies using the Delphi technique to develop their COS, improvements in the reporting of COS, and increased inclusion of patient participants in the COS development process. The development of a reporting guideline and the minimum standards should contribute towards future improvements in development and reporting of COS. This study has also described a first approach to identifying gaps in existing COS, and to priority setting in this area. Important gaps have been identified, on the basis of global burden of disease, and so the development and application of COS in these areas should be considered a priority.

## Supporting Information

S1 PRISMA Checklist PRISMA checklist for content of a systematic review. (DOC)

S1 Table Search strategy. (DOCX)

S2 Table Reason for exclusion at stage 2 (assessment of full text reports). (DOCX)

S3 Table Table of reports included in updated review (n = 25). (DOCX)

S4 Table The methods used to develop core outcome sets (n = 249). (DOCX)

S5 Table Participant groups involved in selecting outcomes in new studies identified in the review update (n = 249). (DOCX)

S6 Table Geographical locations of participants included in the development of each COS (n = 249). (DOCX)

S7 Table Details about COS relevant to 25 conditions with the highest global prevalence. (DOCX)
