# Supplementary material for: Choosing Important Health Outcomes for Comparative Effectiveness Research: An Updated Review and Identification of Gaps
Source: PLoS One. 2016 Dec 14;11(12):e0168403. doi: 10.1371/journal.pone.0168403 (PMC5156438; doi:10.1371/journal.pone.0168403)
Supplement: S2 Table — (DOCX) [file pone.0168403.s003.docx]

**S2 Table.** Reason for exclusion at stage 2 (assessment of full text reports)

| **Reason** | **n** |
| --- | --- |
| Review/overview/discussion only, no outcome recommendations | 51 |
| Irrelevant | 40 |
| Core outcomes/ outcome recommendations not made | 35 |
| Recommendations for clinical management in practice not research | 24 |
| Studies relating to how, rather than which, outcomes should be measured | 18 |
| Systematic reviews of clinical trials | 12 |
| Studies that elicit stakeholder group opinion regarding which outcome domains or outcomes are important | 12 |
| Ongoing studies | 7 |
| Quality indicators – structure and/or process of care only | 6 |
| One outcome/ domain only | 6 |
| Instrument development | 4 |
| Studies reporting the design/ rationale of single trial | 3 |
| HRQL | 3 |
| Describes features of registry | 2 |
| Linked paper to study published in previous review | 2 |
| ICF core set validation | 1 |
| **TOTAL** | **226** |
