# Supplementary material for: Choosing Important Health Outcomes for Comparative Effectiveness Research: An Updated Review and Identification of Gaps
Source: PLoS One. 2016 Dec 14;11(12):e0168403. doi: 10.1371/journal.pone.0168403 (PMC5156438; doi:10.1371/journal.pone.0168403)
Supplement: S3 Table — (DOCX) [file pone.0168403.s004.docx]

**S3 Table.** Table of reports included in updated review (n=25)

| **Study** | **Disease category** | **Disease name** |
| --- | --- | --- |
| Coolsen 2014 [1]** | Cancer | Pancreatic cancer |
| Currie 2015 [2]** | Cancer | Colonic polyps |
| Haeusler 2015 [3]** | Cancer | Fever and Neutropenia |
| Potter 2015 [4]** | Cancer | Breast cancer |
| van den Bos 2014 [5]* | Cancer | Prostate cancer |
| van den Bos 2015 [6]* | Cancer | Prostate cancer |
| Phillips 2015 [7]** | Rheumatology | Chronic musculoskeletal and rheumatologic pain |
| Rendas-Baum 2014 [8]** | Rheumatology | Rheumatoid arthritis |
| Singh 2015 [9]** | Rheumatology | Arthritis |
| Balakrishnan 2015 [10]** | Heart & circulation | Head and neck lymphatic malformation (HNLM) |
| Senni 2014 [11]* | Heart & circulation | Heart failure with preserved ejection fraction |
| Stone 2015 [12]* | Heart & circulation | Mitral regurgitation |
| Harbin Consensus Conference Workshop 2014 [13]* | Pregnancy & childbirth | Infertility |
| van’t Hooft 2015 [14]** | Pregnancy & childbirth | Preterm birth |
| Jones 2012 [15]* | Pregnancy & childbirth | Labour pain |
| Capstick 2014 [16]* | Orthopaedics & trauma | Finger entrapment injuries |
| Chiarotto 2015 [17]** | Orthopaedics & trauma | Low back pain |
| Paul 2014 [18]** | Neurology | Multiple sclerosis |
| Karas 2015 [19]** | Gastroenterology | Acute diarrhoea |
| Billings 2014 [20]** | Kidney disease | Acute kidney injury |
| Bruce 2015 [21]**  Harman 2015 [22]** | Ear, nose & throat | Cleft palate with otitis media effusion |
| Allard 2014 [23]**  Janssens 2014 [24]**  Morris 2015 [25]** | Developmental, psychosocial, & learning problems | Neurodisability |

** Considered outcomes while addressing wider clinical trial design issues*

*** Specifically considered outcome selection and measurement*

**References**

1. Coolsen MM, Clermonts SH, van Dam RM, Winkens B, Malagó M, Fusai GK, et al. Development of a composite endpoint for randomized controlled trials in pancreaticoduodenectomy. World J Surg. 2014;38(6):1468-1475.
2. Currie AC, Cahill R, Delaney CP, Faiz OD, Kennedy RH. International expert consensus on endpoints for full-thickness laparoendoscopic colonic excision. Surg Endosc. 2016;30(4):1497-1502.
3. Haeusler GM, Phillips RS, Lehrnbecher T, Thursky KA, Sung L, Ammann RA. Core outcomes and definitions for pediatric fever and neutropenia research: A consensus statement from an international panel. Pediatr Blood Cancer. 2015; 62:483-489. doi: 10.1002/pbc.25335
4. Potter S, Holcombe C, Ward JA, Blazeby JM. Development of a core outcome set for research and audit studies in reconstructive breast surgery. Br J Surg. 2015;102(11):1360-1371.
5. van den Bos W, Muller BG, Ahmed H, Bangma CH, Barret E, Crouzet S, et al. Focal therapy in prostate cancer: international multidisciplinary consensus on trial design. Eur Urol. 2014;65(6):1078-1083.
6. van den Bos W, Muller BG, de Bruin DM, de Castro Abreu AL, Chaussy C, Coleman JA, et al. Salvage ablative therapy in prostate cancer: International multidisciplinary consensus on trial design. Urol Oncol. 2015;33(11):495.e1-7. doi: 10.1016/j.urolonc.2015.06.015. Epub 2015 Jul 29.
7. Phillips K, Taylor A, Mease PJ, Simon LS, Conaghan PG, Choy EH, et al. Harmonizing pain outcome measures: Results of the pre-OMERACT meeting on partnerships for consensus on patient-important pain outcome domains between the cochrane musculoskeletal group and OMERACT. J Rheumatol. 2015;Aug 1. pii: jrheum.141386. [Epub ahead of print]
8. Rendas-Baum R, Bayliss M, Kosinski M, Raju A, Zwillich SH, Wallenstein GV, et al. Koncz T. Measuring the effect of therapy in rheumatoid arthritis clinical trials from the patient's perspective. Curr Med Res Opin. 2014;30(7):1391-1403.
9. Singh JA, Dohm M, Sprowson AP, Wall PD, Richards BL, Gossec L, et al. Outcome domains and measures in total joint replacement clinical trials: Can we harmonize them? An OMERACT collaborative initiative. J Rheumatol. 2015;42(12):2496-2502.
10. Balakrishnan K, Bauman N, Chun RH, Darrow DH, Grimmer JF, Perkins JA, et al. Standardized outcome and reporting measures in pediatric head and neck lymphatic malformations. Otolaryngol Head Neck Surg. 2015;152(5):948-53. doi: 10.1177/0194599815577602. Epub 2015 Mar 31.
11. Senni M, Paulus WJ, Gavazzi A, Fraser AG, Díez J, Solomon SD, et al. New strategies for heart failure with preserved ejection fraction: the importance of targeted therapies for heart failure phenotypes. Eur Heart J. 2014;35(40):2797-2815. doi: 10.1093/eurheartj/ehu204. Epub 2014 Aug 7.
12. Stone GW, Vahanian AS, Adams DH, Abraham WT, Borer JS, Bax JJ, et al. Clinical trial design principles and endpoint definitions for transcatheter mitral valve repair and replacement: part 1: clinical trial design principles: A consensus document from the mitral valve academic research consortium. Eur Heart J. 2015;36(29):1851-1877. doi: 10.1093/eurheartj/ehv281. Epub 2015 Jul 13.
13. Harbin Consensus Conference Workshop Group; Conference Chairs, Legro RS, Wu X; Scientific Committee, Barnhart KT, Farquhar C, Fauser BC, Mol B. Improving the reporting of clinical trials of infertility treatments (IMPRINT): modifying the CONSORT statement. Hum Reprod. 2014;29(10):2075-82. doi: 10.1093/humrep/deu218. Epub 2014 Sep 12.
14. van ʼt Hooft J^1^, Duffy JM, Daly M, Williamson PR, Meher S, Thom E, et al. A Core Outcome Set for Evaluation of Interventions to Prevent Preterm Birth. Obstet Gynecol;127(1):49-58. doi: 10.1097/AOG.0000000000001195.
15. Jones L, Othman M, Dowswell T, Alfirevic Z, Gates S, Newburn M, et al. Pain management for women in labour: an overview of systematic reviews. Cochrane Database Syst Rev. 2012; (3):CD009234. doi: 10.1002/14651858.CD009234.pub2.
16. Capstick R, Giele H. Interventions for treating fingertip entrapment injuries in children. Cochrane Database Syst Rev. 2014;(4):CD009808. DOI: 10.1002/14651858.CD009808.pub2.
17. Chiarotto A, Deyo RA, Terwee CB, Boers M, Buchbinder R, Corbin TP, et al. Core outcome domains for clinical trials in non-specific low back pain. Eur Spine J. 2015;24(6):1127-42. doi: 10.1007/s00586-015-3892-3. Epub 2015 Apr 5.
18. Paul L, Coote S, Crosbie J, Dixon D, Hale L, Holloway E, et al. Core outcome measures for exercise studies in people with multiple sclerosis: recommendations from a multidisciplinary consensus meeting. Mult Scler. 2014;20(12):1641-1650. doi: 10.1177/1352458514526944. Epub 2014 Mar 17.
19. Karas J, Ashkenazi S, Guarino A, Lo Vecchio A, Shamir R, Vandenplas Y, et al. A core outcome set for clinical trials in acute diarrhoea. Arch Dis Child. 2015;100(4):359-63. doi: 10.1136/archdischild-2014-307403. Epub 2014 Nov 20.
20. Billings FT, Shaw AD. Clinical trial endpoints in acute kidney injury. Nephron. 2014;127(1-4):89-93.
21. Bruce I, Harman N, Williamson P, Tierney S, Callery P, Mohiuddin S, et al. The management of otitis media with effusion in children with cleft palate (mOMEnt): A feasibility study and economic evaluation. Health Technol Assess. 2015;19(68):1-374. doi: 10.3310/hta19680.
22. Harman NL, Bruce IA, Kirkham JJ, Tierney S, Callery P, O'Brien K, et al. The Importance of Integration of Stakeholder Views in Core Outcome Set Development: Otitis Media with Effusion in Children with Cleft Palate. PLoS ONE. 2015;10(6):e0129514. doi:10.1371/journal.pone.0129514
23. Allard A, Fellowes A, Shilling V, Janssens A, Beresford B, Morris C. Key health outcomes for children and young people with neurodisability: qualitative research with young people and parents. BMJ Open. 2014;4:e004611.
24. Janssens A, Williams J, Tomlinson R, Logan S, Morris C. Health outcomes for children with neurodisability: what do professionals regard as primary targets? Arch Dis Child. 2014;99(10):927–32.
25. Morris C, Janssens A, Shilling V, Allard A, Fellowes A, Tomlinson R, et al. Meaningful health outcomes for paediatric neurodisability: Stakeholder prioritisation and appropriateness of patient reported outcome measures. Health Qual Life Outcomes. 2015;13:87. doi: 10.1186/s12955-015-0284-7.
