# Supplementary material for: Choosing Important Health Outcomes for Comparative Effectiveness Research: An Updated Review and Identification of Gaps
Source: PLoS One. 2016 Dec 14;11(12):e0168403. doi: 10.1371/journal.pone.0168403 (PMC5156438; doi:10.1371/journal.pone.0168403)
Supplement: S4 Table — (DOCX) [file pone.0168403.s005.docx]

**S4 Table.** The methods used to develop core outcome sets (n=249)

| **Main methods** | **Original review**  **n** | **Update review 1**  **n** | **Update review 2**  **n** |
| --- | --- | --- | --- |
| **Semi-structured group discussion only** | **57** | **2** | **2** |
| *Workshop* | *22* | *1* | *1* |
| *Meeting (meeting, colloquium, conference where not described as consensus development conference)* | *32* | *1* | *1* |
| *Round table discussion* | *3* |  |  |
| **Unstructured group discussion only**  *Descriptions include task force, work group, working group/party, committee, board, panel* | **18** |  |  |
| **Consensus development conference only** | **12** |  | **1** |
| **Literature/systematic review only** | **11** | **5** | **2** |
| **Delphi only** | **6** | **2** | **2** |
| **Survey only** | **3** |  |  |
| **NGT only** | **1** |  |  |
| **Mixed methods *(see descriptions below*)** | **74** | **18** | **14** |
| ***Delphi + another method(s)*** | ***23*** | ***7*** | ***10*** |
| *Literature/systematic review* | *5* | *1* | *5* |
| *Literature/systematic review + NGT* | *4* |  |  |
| *NGT* | *4* |  |  |
| *Literature/systematic review + semi-structured group discussion (meeting/workshop)* | *3* | *2* | *4* |
| *Semi-structured discussion (meeting/workshop)* | *2* | *1* |  |
| *Literature/systematic review + survey* | *1* |  |  |
| *Literature/systematic review + consensus conference* | *1* |  |  |
| *Literature/systematic review + semi-structured discussion (meeting) + survey* | *1* |  |  |
| *Literature/systematic review + semi-structured discussion (meeting) + focus group(s) + workshop* | *1* |  |  |
| *Semi-structured group discussion (meeting) + survey* | *1* |  |  |
| *Literature/systematic review + unstructured group discussion* |  | *1* |  |
| *Literature/systematic review + focus group(s) + interviews* |  | *1* |  |
| *NGT + Focus groups* |  | *1* |  |
| *Literature/systematic review + semi-structured discussion (meeting) + interviews + focus group(s)* |  |  | *1* |
| ***Semi-structured group discussion (listed which method) + another method(s)*** | ***31*** | ***7*** | ***4*** |
| *Meeting + literature/systematic review* | *13* | *4* | *1* |
| *Workshop + literature/systematic review* | *4* |  |  |
| *Meeting/Workshop + survey + literature/systematic review* | *3* | *1* |  |
| *Workshop and meeting* | *2* |  |  |
| *Workshop/meetings + web-based consultation* | *2* |  |  |
| *Round table discussion + literature/systematic review* | *2* |  |  |
| *Workshop, literature/systematic review* | *1* |  |  |
| *Meeting + focus group(s) + survey* | *1* |  |  |
| *Meeting + survey* | *1* |  |  |
| *Workshop and meetings + NGT* | *1* |  |  |
| *Workshop + literature/systematic review + NGT* | *1* |  |  |
| *Meeting + literature/systematic review + dataset analysis* |  | *1* |  |
| *Meeting + literature/systematic review + survey + email discussions* |  | *1* |  |
| *Meeting + literature/systematic review + NGT* |  |  | *1* |
| *Meeting + use of ICF* |  |  | *1* |
| *Meeting + prioritisation task* |  |  | *1* |
| ***Consensus development conference + another method(s)*** | ***7*** |  |  |
| *Literature/systematic review* | *3* |  |  |
| *Survey* | *1* |  |  |
| *NGT* | *1* |  |  |
| *Meeting(s)* | *1* |  |  |
| *Literature/systematic review + survey + meeting* | *1* |  |  |
| ***Literature/systematic review + another method(s)*** | ***10*** | ***4*** |  |
| *Unstructured group discussion* | *5* | *1* |  |
| *Public presentation and debate* | *2* |  |  |
| *Survey* | *1* |  |  |
| *Survey + open discussion* | *1* |  |  |
| *NGT* | *1* | *2* |  |
| *Interviews* |  | *1* |  |
| ***NGT + another method(s)*** | ***2*** |  |  |
| *Survey + interview* | *1* |  |  |
| *Survey* | *1* |  |  |
| ***Focus group + rating exercise*** | ***1*** |  |  |
| **No methods described** | **16** | **2** | **1** |
