# Supplementary material for: Choosing Important Health Outcomes for Comparative Effectiveness Research: An Updated Review and Identification of Gaps
Source: PLoS One. 2016 Dec 14;11(12):e0168403. doi: 10.1371/journal.pone.0168403 (PMC5156438; doi:10.1371/journal.pone.0168403)
Supplement: S5 Table — (DOCX) [file pone.0168403.s006.docx]

**S5 Table.** Participant groups involved in selecting outcomes in new studies identified in the review update (n=249)

| **Participants category** | **Sub-category (not mutually exclusive)** | **Frequency of participants** | | |
| --- | --- | --- | --- | --- |
|  |  | **Original review**  **n** | **Update review 1**  **n** | **Update review 2**  **n** |
| **Clinical experts** | | **173/198** | **21/29** | **18/22** |
|  | Clinical experts | 88 | 15 | 17 |
|  | Clinical research expertise | 67 | 10 | 9 |
|  | Clinical trialists/Members of a clinical trial network | 10 | 2 |  |
|  | Others with assumptions* | 54 |  |  |
| **Patient representatives** | | **31/198** | **13/29** | **11/22** |
|  | Patients | 20 | 11 | 7 |
|  | Carers | 7 | 1 | 3 |
|  | Patient support group representatives | 9 | 1 | 4 |
|  | Service users | 2 |  |  |
| **Non-clinical research experts** | | **54/198** | **10/29** | **9/22** |
|  | Researchers | 26 | 4 | 4 |
|  | Statisticians | 20 | 4 | 3 |
|  | Epidemiologists | 11 | 2 | 1 |
|  | Academic research representatives | 4 |  |  |
|  | Methodologists | 6 | 4 | 2 |
|  | Economists | 3 |  | 1 |
| **Authorities** | | **40/198** | **5/29** | **3/22** |
|  | Regulatory agency representatives | 31 | 4 | 3 |
|  | Governmental agencies | 12 | 1 |  |
|  | Policy makers | 4 | 1 |  |
|  | Charities | 1 |  |  |
| **Industry representatives** | | **32/198** | **4/22** | **3/22** |
|  | Pharmaceutical industry representatives | 29 | 3 | 3 |
|  | Device manufacturers | 2 | 1 |  |
|  | Biotechnology company representatives | 1 |  |  |
| **Others** | | **72/198** | **2/29** | **1/22** |
|  | Ethicists | 1 |  |  |
|  | Journal editors | 2 |  | 1 |
|  | Funding bodies |  | 1 |  |
|  | Yoga therapists/instructors |  | 1 |  |
|  | Others** (besides known participants) | 15 |  |  |
|  | Others with assumptions* | 54 |  |  |
| **No details given** | | **24/198** | **7/29** | **4/22** |

** 54 studies with clinical input but unclear about involvement of other stakeholders*

*** Workshop/meeting participants (*5), subcommittee/committee (*2), guidelines panel, military personnel, moderator and audience, representatives from EORTC, members with expertise in information technologies, informatics, clinical registries, data-standards development, expertise in vaccine safety, malaria control and representatives from funding agencies/registration authorities, and donor organisation, members of the Rheumatology Section of the American Academy of Pediatrics, the Pediatric Section of the ACR, and the Arthritis Foundation, the diagnostic radiology and basic science communities, and from individuals conversant with functional and quality of life (QOL) assessments, comparative effectiveness research, and cost/ benefit analysis*
