# Supplementary material for: Choosing Important Health Outcomes for Comparative Effectiveness Research: An Updated Review and Identification of Gaps
Source: PLoS One. 2016 Dec 14;11(12):e0168403. doi: 10.1371/journal.pone.0168403 (PMC5156438; doi:10.1371/journal.pone.0168403)
Supplement: S6 Table — (DOCX) [file pone.0168403.s007.docx]

**S6 Table.** Geographical locations of participants included in the development of each COS (n=249)

| **Continents** | **n (%)** | | | **Median and range of number of countries** | | |
| --- | --- | --- | --- | --- | --- | --- |
|  | **Original review** | **Update review 1** | **Update review 2** | **Original review** | **Update review 1** | **Update review 2** |
| North America, Europe | 56^1*^ (28) | 7^4^ (24) | 3^4^ (14) | 4, 2-25 | 6, 2-14 | 3, 3-6 |
| North America | 44^2^ (22) | 6^6^ (21) | 3^5^ (14) | 1, 1-2 | 1 | 1, 1 |
| Europe | 32^3^ (16) | 9^5^ (31) | 4^5^ (18) | 2, 1-14 | 1 | 1, 1 |
| North America, Europe, Australasia | 13^4^* (7) | 2 (7) | 3 (14) | 7, 3-25 | 5, 3-6 | 5, 4-5 |
| North America, Europe, Asia | 11^5^ (6) | 1 (4) | 3 (14) | 9, 5-14 | 10 | 11, 8-12 |
| North America, Europe, Australasia, Asia | 10^4^ (5) |  | 2^5^ (9) | 11, 6-15 |  | 11, 10-11 |
| North America, Europe, Australasia, Asia, South America | 10^4^* (5) |  |  | 16, 5-21 |  |  |
| North America, Europe, Australasia, Asia, South America, Africa | 4 (2) | 1 (4) | 1 (5) | 26, 8-46 | 33 | 28 |
| North America, Europe, Australasia and Africa | 3^5^** (2) |  |  | 8, 3-17 |  |  |
| North America, Europe, South America | 2 (1) | 1^5^ (4) |  | 10, 9-11 | 3 |  |
| North America, Europe, Asia, South America | 2 (1) | 1 (4) |  | 11, 7-15 | 6 |  |
| North America, Australasia | 2 (1) |  |  | 3, 3 |  |  |
| Australasia | 1 (<1) | 1 (4) |  | 2 | 2 |  |
| Europe, Australasia | 1 (<1) |  | 1 (5) | 2 |  | 2 |
| North America, Europe, Australasia, South America | 1 (<1) |  | 1 (5) | 11 |  | 15 |
| North America, Europe, Africa | 1 (<1) |  |  | 10 |  |  |
| North America, Asia, South America, Africa | 1 (<1) |  |  | 5 |  |  |
| North America, Europe, South America, Africa | 1 (<1) |  |  | 7 |  |  |
| North America, Europe, Australasia, Asia, Africa | 1 (<1) |  |  | 15 |  |  |
| North America, Europe, Australasia, South America, Africa | 1 (<1) |  |  | 8 |  |  |
| North America, Europe, Asia, South America, Africa | 1 (<1) |  |  | 18 |  |  |
| North America, Europe, Asia, Africa |  |  | 1 (5) |  |  | 28 |

Locations for participants other than the lead contact/participating authors were not provided (^1^ – 15 studies, ^2^ – 9 studies, ^3^ – 7 studies, ^4^ – 2 studies, ^5^ – 1 study, ^6^ – 3 studies)

** In 6 studies, OMERACT participants' information was extracted from the introductory paper*

*** In 1 study, participants’ location was based on where they had graduated from*
