# Supplementary material for: Choosing Important Health Outcomes for Comparative Effectiveness Research: An Updated Review and Identification of Gaps
Source: PLoS One. 2016 Dec 14;11(12):e0168403. doi: 10.1371/journal.pone.0168403 (PMC5156438; doi:10.1371/journal.pone.0168403)
Supplement: S7 Table — (DOCX) [file pone.0168403.s008.docx]

**S7 Table.** Details about COS relevant to 25 conditions with the highest global prevalence

| **Condition** | **Year of publication** | **Scope** | | | **Stakeholders involved** | | | **Countries involved** |
| --- | --- | --- | --- | --- | --- | --- | --- | --- |
|  |  | **Population** | **Interventions** | **Setting** | **Clinical experts** | **Patients/ public** | **Other** |  |
| Asymptomatic permanent caries | Pitts 2004 [1] | Age (not specified); Sex (either) | Oral care products | Clinical trials | Workshop participants – leading group of experts |  |  | UK, USA |
|  | Schwendicke (Ongoing) [2] | Age (adults and children); Sex (either) | Behavioural, Drug, Surgery | Clinical trials | Dentists, Clinical researchers | Patients | Health economists, Statisticians, Policy makers, Industry representatives | Germany, UK |
| Individuals with recurrent tension-type headaches | Schoenen 1995 [3] Bendtsen 2010 [4]  (linked studies) | Age (adults); Sex (either) | Drug | Clinical trials | Clinical trials subcommittee of the International headache society (no details about discipline) |  |  | Denmark, Belgium |
|  | Penzien 2005 [5]  Penzien 2005 [6]  Andrasik 2005 [7]  (linked studies) | Age (adults, but makes recommendations for children);  Sex (either) | Behavioural therapies | Clinical trials/ research | American Headache Society's Behavioural Clinical Trials Workgroup, headache researchers |  |  | USA |
| Iron-deficiency anaemia | No published/ ongoing COS | | | | | | | |
| Glucose-6-phosphate dehydrogenase deficiency trait | No published/ ongoing COS | | | | | | | |
| Age-related and other hearing loss | No published/ ongoing COS | | | | | | | |
| Asymptomatic genital herpes | No published/ ongoing COS | | | | | | | |
| Individuals with recurrent migraine | Tfelt-Hansen 1991 [8]  Tfelt-Hansen 2001 [9]  Tfelt-Hansen 2012 [10]  (linked studies) | Age (adults, but provides comments for children);  Sex (either) | Drug | Clinical trials | International Headache Society Clinical Trials Subcommittee (names and locations of members are provided, but no disciplines) |  |  | Belgium, UK, Denmark, Spain, France, Sweden, Italy, Germany, USA |
|  | Smelt 2014 [11] | Age (adult);  Sex (either) | Medication - drug | Effectiveness studies |  | Patients |  | Netherlands |
| Ascariasis | No published/ ongoing COS | | | | | | | |
| Fungal skin diseases | No published/ ongoing COS | | | | | | | |
| Acne vulgaris | Thiboutot (Ongoing) [12] | Age (14-45); Sex (either) | Any | Clinical trials | Clinical experts | Patients | Device manufacturers | USA, Canada, UK |
|  | Clark (Ongoing) [13]  (acne scarring) | Age (adults); Sex (either) | Any | Not reported | Clinical experts | Patients, Families | Governmental agencies, Methodologists, Pharmaceutical industry representatives | USA |
| Uncorrected refractive error | No published/ ongoing COS | | | | | | | |
| Low back pain | Deyo 1998 [14] | Age (not specified); Sex (either) | Not specified | Clinical research | Group of investigators (back pain researchers) |  |  | USA, Canada, Netherlands, UK, Finland |
|  | Devogelaer 2003 [15] | Age (not specified); Sex (either) | Drugs | Efficacy studies | Clinical researchers |  | Basic science researchers, Representatives of national and European drug licensing authorities, Members of pharmaceutical industry | Belgium, France, Spain, Austria, Germany,  Portugal |
|  | Chiarotto 2015 [16] | Age (adults); Sex (either) | Any | Clinical trials | Healthcare providers, Healthcare researchers | Patients, Patient representatives |  | Netherlands, USA, Spain, Australia, Brazil, UK, Norway |
| Periodontal diseases | Page 1992 [17] | Age (not specified); Sex (either) | Any | Clinical trials | Clinical experts* |  |  | USA |
|  | Imrey 1994 [18] | Age (adults); Sex (either) | Non-surgical treatment | Clinical trials (scaling and root planning) and non-surgical treatment | Dental researchers |  | Task Force on Design and Analysis of Dental and Oral Research, Statistician, Government members of the periodontal research community, Industry members of the periodontal research community | USA |
|  | Lightfoot 2005 [19] (Chronic periodontitis - anterior teeth) | Age (not specified); Sex (either) | Not specified | Clinical trials | American Academy of Periodontology, or in leadership positions in organized dentistry |  | Academic research reps, Military Personnel | USA |
|  | Lightfoot 2005 [20] (Chronic periodontitis - posterior teeth) | Age (not specified); Sex (either) | Not specified | Clinical trials | Periodontists |  |  | USA |
|  | Glenny Planning [21] | Age (adults); Sex (either) | Drug, Physical, Surgery | Clinical trials | Clinical experts | Patients |  | UK |
| Other skin and subcutaneous diseases | Gonzales 2011 [22]  Eleftheriadou 2012 [23]  Eleftheriadou 2015 [24] (linked studies: Vitiligo) | Age (adults); Sex (either) | Any | Clinical trials | Clinicians/ dermatologists and other healthcare professionals | Patients, Carers, Patient representatives, | Coordinator of the vitiligo priority-setting partnership at the Centre of Evidence-Based Dermatology at the University of Nottingham, Statistician | UK, Spain, Belgium, USA, Japan, Egypt, Singapore,  Morocco, France, Brazil  Taiwan, Saudi Arabia, Korea, India, Italy |
|  | Olliaro 2013 [25] (Cutaneous leishmaniasis) | Age (adults and children);  Sex (either) | Not specified | Clinical trials | Clinical experts* |  |  | Switzerland, UK, USA, Luxemburg, France |
|  | Simpson 2013 [26]  (Vulval skin condition) | Age (not specified); Sex (female) | Any | Clinical practice and research | Clinical experts* |  |  | UK |
|  | Tang (Ongoing) [27] (Melasma) | Age (adults); Sex (female) | Any | Not reported | Clinical experts | Patients, Families | Governmental agencies, Methodologists, Pharmaceutical industry representatives | USA |
|  | Shokeen (Ongoing) [28] (Post-Inflammatory Hyperpigmentation) | Age (adults); Sex (female) | Any | Not reported | Clinical experts | Patients, Families | Governmental agencies, Methodologists, Pharmaceutical industry representatives | USA |
|  | Vasic (Ongoing) [29] (Actinic Keratosis) | Age (adults); Sex (female) | Any | Not reported | Clinical experts | Patients, Families | Governmental agencies, Methodologists, Pharmaceutical industry representatives | USA |
|  | Colavincenzo (Ongoing) [30] (Alopecia) | Age (adults); Sex (female) | Any | Not reported | Clinical experts | Patients, Families | Governmental agencies, Methodologists, Pharmaceutical industry representatives | USA |
|  | Prinsen (Planning) [31] (Melanoma) | Age (adults); Sex (either) | Not reported | Clinical trials | Clinical experts | Patients, Patient/ support group representatives | Epidemiologists  Journal editors, Methodologists, Pharmaceutical industry representatives, Policy makers, Regulatory agency representatives, Researchers | The Netherlands, USA, Germany |
| Trichuriasis | No published/ ongoing COS | | | | | | | |
| Asymptomatic deciduous caries | Pitts 2004 [1] | Age (not specified); Sex (either) | Oral care products | Clinical trials | Workshop participants - group of experts* |  |  | UK, USA |
|  | Schwendicke (Ongoing) [2] | Age (adults and children); Sex (either) | Behavioural, Drug, Surgery | Clinical trials | Dentists, Clinical researchers | Patients | Health economists, Statisticians, policy makers, Industry representatives | Germany, UK |
| Hookworm disease | No published/ ongoing COS | | | | | | | |
| Diabetes mellitus | Ashfaq (Ongoing) [32] (Type II diabetes) | Age (adults); Sex (either) | Drug | Not reported |  |  | Not reported | UK |
|  | Byrne (Ongoing) [33] (Type I diabetes) | Age (young adults);  Sex (either) | Any | Not reported | Clinical experts | Patients, Families | Researchers | Ireland, UK, Denmark, Canada, Australia |
| Neck pain | No published/ ongoing COS | | | | | | | |
| Genital prolapse | Toozs-Hobson 2012 [34] (Pelvic organ prolapse) | Age (not specified); Sex (female) | Surgical procedures | Research | Committee of International Urogynecological Association (IUGA) and International Continence Society (ICS), a joint working group and expert external referees* |  |  | UK, Australia, Greece, USA, Belgium |
| Malaria parasitaemia, anaemia, or chronic sequelae | No published/ ongoing COS** | | | | | | | |
| Glucose-6-phosphate dehydrogenase deficiency | No published/ ongoing COS | | | | | | | |
| Dermatitis | Schmitt 2007 [35]  Schmitt 2010 [36]  Schmitt 2011 [37]  Schmitt 2012 [38]  (linked studies: Atopic eczema) | Age (adults and children); Sex (either) | Not specified | Clinical trials and clinical record keeping (separate sets) | Dermatology experts | Patients, Carers | Methodologists, pharmaceutical industry representatives, journal editors, and regulatory agency representatives | Germany  Netherlands  UK, Brazil, Israel, USA, Japan, France, Australia, Sweden |
|  | Beeckman (Ongoing) [39] (Incontinence-associated dermatitis) | Age (adults and children); Sex (either) | Skin care | Clinical research | Clinical experts | Patients | Researchers | Belgium, Germany |
|  | Iyengar (Ongoing) [40] (Rosacea) | Age (adults); Sex (female) | Any | Not reported | Clinical experts | Patients, Families | Governmental agencies, Methodologists, Pharmaceutical industry representatives | USA |
| Chronic obstructive pulmonary disease | Task Group on Mucoactive Drugs 1994 [41] | Age (not specified); Sex (either) | Drugs | Clinical efficacy during  phase III studies | Experts* |  |  | Spain, Italy, France, UK, Germany, USA |
|  | Cazzola 2008 [42] | Age (not specified); Sex (either) | Pharmacological - drugs | Clinical trials | Experts in COPD trials and in specific COPD outcomes* |  |  | UK, Italy, USA, Spain, Netherlands |
| Chronic hepatitis B infection | Sun (Ongoing) [43] (Chinese medicine in treatment of chronic hepatitis) | Age (not specified); Sex (either) | Complementary and alternative medicine therapy) | Clinical trials | Clinical experts | Patients | Journal editors | China |

*Stakeholders involved are assumed to be clinical experts, on account of the descriptions or affiliations provided

** Only COS known are for vaccine trials

**References**

[1] Pitts NB, Stamm JW. International Consensus Workshop on Caries Clinical Trials (ICW-CCT)--final consensus statements: agreeing where the evidence leads. *J Dent Res*. 2004;83:C125-128.

[2] Schwendicke F, Innes N, Lamont T. Outcomes in Trials for Management of Caries Lesions (OuTMaC). <http://www.comet-initiative.org/studies/details/694>

[3] Schoenen J. Guidelines for trials of drug treatments in tension-type headache. First Edition: International Headache Society Committee on Clinical Trials. *Cephalgia*. 1995;15(3):165-179.

[4] Bendtsen L, Bigal ME, Cerbo R, Diener HC, Holroyd K, Lampl C, et al. Guidelines for controlled trials of drugs in tension-type headache: second edition. *Cephalalgia.* 2010;30(1):1-16.

[5] Penzien DB, Andrasik F, Freidenberg BM, Houle TT, Lake AE 3rd, Lipchik GL, et al. Guidelines for trials of behavioral treatments for recurrent headache, first edition: American Headache Society Behavioral Clinical Trials Workgroup. *Headache.* 2005;45(Suppl 2):S110-132.

[6] Penzien DB. Guidelines for Trials of Behavioral Treatments for Recurrent Headache: Purpose, Process, and Product. *Headache.* 2005;45(Suppl 2):S87-S89).

[7] Andrasik F, Lipchik GL, McCrory DC, Wittrock DA. Outcome Measurement in Behavioral Headache Research: Headache Parameters and Psychosocial Outcomes. *Headache.* 2005;45(5):429-437.

[8] Tfelt-Hansen P. Guidelines for controlled trials of drugs in migraine: first edition. International Headache Society Committee on Clinical Trials in Migraine. *Cephalalgia.* 1991;11(1-12).

[9] Tfelt-Hansen P, Block G, Dahlöf C, Diener HC, Ferrari MD, Goadsby PJ, et al. Guidelines for controlled trials of drugs in migraine: second edition. *Cephalalgia.* 2000;20(9):765-786.

[10] Tfelt-Hansen P, Pascual J, Ramadan N, Dahlöf C, D'Amico D, Diener HC, et al. Guidelines for controlled trials of drugs in migraine: third edition. A guide for investigators. *Cephalalgia.* 2012;32(1):6-38.

[11] Smelt AF, Louter MA, Kies DA, Blom JW, Terwindt GM, van der Heijden GJ, et al. What do patients consider to be the most important outcomes for effectiveness studies on migraine treatment? Results of a Delphi study. *PLoS One.* 2014;9(6):e98933.

[12] Thiboutot D, Tan J, Layton A. Development of Clinical Trials Outcome Instruments for Acne Vulgaris. <http://www.comet-initiative.org/studies/details/358>

[13] Clark M, Samuelson E, Schaeffer M, Alam M. Core Outcome Set for Acne Scarring. <http://www.comet-initiative.org/studies/details/754>

[14] Deyo RA, Battie M, Beurskens AJ, Bombardier C, Croft P, Koes B, et al. Outcome measures for low back pain research. A proposal for standardized use. *Spine*. 1998;23(18):2003-2013.

[15] Devogelaer JP, Dreiser RL, Abadie E, Avouac B, Bouvenot G, Carbonell Abello J, et al. Guidelines for clinical studies assessing the efficacy of drugs for the management of acute low back pain. *Clin Exp Rheumatol*. 2003;21(6):691-694.

[16] Chiarotto A, Deyo RA, Terwee CB, Boers M, Buchbinder R, Corbin TP, et al. Core outcome domains for clinical trials in non-specific low back pain. *Eur Spine J*. 2015;24(6):1127-1142.

[17] Page RC, DeRouen TA. Design issues specific to studies of periodontitis. *J Periodontal Res.* 1992;27(4 Pt 2):395-404;discussion 412-416.

[18] Imrey PB, Chilton NW, Pihlstrom BL, Proskin HM, Kingman A, Listgarten MA, et al. Proposed guidelines for American Dental Association acceptance of products for professional, non-surgical treatment of adult periodontitis. Task Force on Design and Analysis in Dental and Oral Research. *J Periodontal Res.* 1994;29(5):348-360.

[19] Lightfoot WS, Hefti A, Mariotti A. Using a Delphi panel to survey criteria for successful periodontal therapy in posterior teeth. *J Periodontol.* 2005;76(9):1502-1507.

[20] Lightfoot WS, Hefti A, Mariotti A. Using a Delphi panel to survey criteria for successful periodontal therapy in anterior teeth. *J Periodontol.* 2005;76(9):1508-1512.

[21] Glenny A-M, Worthington H, Walsh T, Burnside G. Core outcome measures and selective outcome reporting in randomised controlled trials for the prevention and treatment of periodontal disease. <http://www.comet-initiative.org/studies/details/265>

[22] González U, Whitton M, Eleftheriadou V, Pinart M, Batchelor J, Leonardi-Bee J. Guidelines for designing and reporting clinical trials in vitiligo. *Arch Dermatol.* 2011;147(12):1428-1436.

[23] Eleftheriadou V, Thomas KS, Whitton ME, Batchelor JM, Ravenscroft JC. Which outcomes should we measure in vitiligo? Results of a systematic review and a survey among patients and clinicians on outcomes in vitiligo trials. *Br J Dermatol.* 2012;167(4):804-814.

[24] Eleftheriadou V, Thomas K, van Geel N, Hamzavi I, Lim H, Suzuki T, et al. Developing core outcome set for vitiligo clinical trials: international e-Delphi consensus. *Pigment Cell Melanoma Res.* 2015;28(3):363-369.

[25] Olliaro P1, Vaillant M, Arana B, Grogl M, Modabber F, Magill A, et al. Methodology of clinical trials aimed at assessing interventions for cutaneous leishmaniasis. *PLoS Negl Trop Dis.* 2013;7(3):e2130.

[26] Simpson RC1, Thomas KS, Murphy R. Outcome measures for vulval skin conditions: a systematic review of randomized controlled trials. *Br J Dermatol.* 2013;169(3):494-501.

[27] Tang J, Samuelson E, Schaeffer M, Alam M. Core Outcome Set for Melasma. <http://www.comet-initiative.org/studies/details/752>

[28] Shokeen D, Samuelson E, Schaeffer M, Alam M. Core Outcome Set for Post-Inflammatory Hyperpigmentation (PIH). <http://www.comet-initiative.org/studies/details/753>

[29] Vasic J, Samuelson E, Goldberg L, Alam M. Core Outcome Set for Actinic Keratosis. <http://www.comet-initiative.org/studies/details/756>

[30] Colavincenzo M, Schlessinger D, Iyengar S, Samuelson E, Schaeffer M, Alam M. Core Outcome Set for Hair Loss/Non-Scarring Alopecia. <http://www.comet-initiative.org/studies/details/759>

[31] Prinsen CAC, van Akkooi A, Bekkenk MW, Boers M, Chauhan C, Deckert S, et al. Developing a Core Outcome Set (COS) for melanoma trials. <http://www.comet-initiative.org/studies/details/783>

[32] Ashfaq K, Walsh T, Glenny A-M. Identification and development of a core set of outcome measures for the treatment of type 2 diabetes in adults. <http://www.comet-initiative.org/studies/details/364>

[33] Byrne M, O’Connell A, Egan A, Dinneen S, Hynes L, Holt R, et al. Developing a core outcome set (COS) for clinical studies of young adults with type 1 diabetes mellitus. <http://www.comet-initiative.org/studies/details/792>

[34] Toozs-Hobson P, Freeman R, Barber M, Maher C, Haylen B, Athanasiou S, et al. An International Urogynecological Association (IUGA)/International Continence Society (ICS) joint report on the terminology for reporting outcomes of surgical procedures for pelvic organ prolapse. *Int Urogynecol J.* 2012;23(5):527-535.

[35] Schmitt J, Langan S, Williams HC; European Dermato-Epidemiology Network. What are the best outcome measurements for atopic eczema? A systematic review. *J Allergy Clin Immunol*. 2007;120(6):1389-1398.

[36] Schmitt J, Williams H, HOME Development Group. Harmonising Outcome Measures for Eczema (HOME). Report from the First International Consensus Meeting (HOME 1), 24 July 2010, Munich, Germany. *Br J Dermatol*. 2010;163(6):1166-1168.

[37] Schmitt J, Langan S, Stamm T, Williams HC; Harmonizing Outcome Measurements in Eczema (HOME) Delphi panel. Core outcome domains for controlled trials and clinical recordkeeping in eczema: international multiperspective Delphi consensus process. *J Invest Dermatol*. 2011;131(3):623-630.

[38] Schmitt J, Spuls P, Boers M, Thomas K, Chalmers J, Roekevisch E, et al. Towards global consensus on outcome measures for atopic eczema research: results of the HOME II meeting. *Allergy*. 2012;67(9):1111-1117.

[39] Beeckman D, Kottner J. Developing a Core Outcome Set for Incontinence-Associated Dermatitis (IAD) Research. <http://www.comet-initiative.org/studies/details/383>

[40] Iyengar S, Samuelson E, Schaeffer M, Alam M. Core Outcome Set for Rosacea. <http://www.comet-initiative.org/studies/details/758>

[41] Recommendations for guidelines on clinical trials of mucoactive drugs in chronic bronchitis and chronic obstructive pulmonary disease. Task Group on Mucoactive Drugs. *Chest.* 1994;106(5):1532-1537.

[42] Cazzola M, MacNee W, Martinez FJ, Rabe KF, Franciosi LG, Barnes PJ, et al. Outcomes for COPD pharmacological trials: from lung function to biomarkers. *Eur Respir J.* 2008;31(2):416-469.

[43] Sun Y, Yu C, He L, Fan J. Core Outcome Sets of integrity of modern and Traditional Chinese Medicine on treatment of chronic Hepatitis B. <http://www.comet-initiative.org/studies/details/669>
